# The use of attention-deficit hyperactivity disorder medications in cardiac disease

**DOI:** 10.3389/fnins.2022.1020961

**Published:** 2022-10-19

**Authors:** Constantin-Cristian Topriceanu, James C. Moon, Gabriella Captur, Bhathika Perera

**Affiliations:** ^1^Barnet, Enfield and Haringey Mental Health Trust, London, United Kingdom; ^2^UCL Institute of Cardiovascular Science, University College London, London, United Kingdom; ^3^UCL MRC Unit for Lifelong Health and Ageing, University College London, London, United Kingdom; ^4^Cardiac MRI Unit, Barts Heart Centre, London, United Kingdom; ^5^Centre for Inherited Cardiovascular Diseases, Great Ormond Street Hospital, London, United Kingdom; ^6^Department of Cardiology, Centre for Inherited Heart Muscle Conditions, The Royal Free Hospital, London, United Kingdom

**Keywords:** ADHD (attention deficit and hyperactivity disorder), cardiovascular disease, stimulant medications for ADHD, non-stimulant medications for ADHD, electrocardiogram (ECG), blood pressure, cardiomyopathies, aortopathies

## Abstract

Attention-deficit hyperactivity disorder (ADHD) is a neurodevelopmental disorder with onset usually in childhood characterized by inattention, impulsivity, and hyperactivity causing a functional impairment. Untreated ADHD, or treatment delay is associated with adverse outcomes and poor quality of life. Although conservative management strategies such as behavioral and psychological interventions are important, pharmacological treatment has a strong evidence base with improved outcomes. ADHD medications are broadly divided into stimulant and non-stimulant medications. Stimulant medications are generally more effective than non-stimulants. Cardiovascular safety of ADHD medication has been a matter of debate for decades. Treatment guidelines advise the careful consideration of risks and benefits in people with cardiovascular diseases such as congenital heart disease or cardiomyopathy. Although stimulants can increase systemic blood pressure and heart rate, no significant associations were found between their use and serious cardiovascular events. Concerns regarding QT effects and attendant sudden cardiac death risks deter clinicians from initiating much-needed ADHD medications in patients with heart disease. This overly cautious approach is potentially depriving low-risk individuals from significant benefits associated with timely ADHD drug treatment. This review discusses the cardiovascular risks reportedly associated with ADHD medications, the evidence base for their safe usage in persons with established cardiovascular disease, and highlights future research directions.

## Introduction

Attention-deficit hyperactivity disorder (ADHD) is a neurodevelopmental disorder (NDD) affecting around 5% of the population (Polanczyk and Rohde, 2007). Diagnosis of ADHD includes the presence of core symptoms of inattention, and/or hyperactivity/impulsivity. Symptoms manifest from early childhood in all cases and cause a functional impairment in personal, social, academic or occupational functioning (American Psychiatric Association., 2013). Treatment of ADHD is associated with improvements in core symptoms of ADHD along with a reduction in functional impairment in different domains of life. Although psychoeducation and behavioral strategies play a vital role in the management of ADHD, pharmacological treatments have a strong evidence base for their effectiveness (Brown et al., 2018).

It is hypothesized that ADHD is associated with a decrease in pre-frontal cortex and limbic system size and function (Arnsten, 2009). In addition, it is characterized by decreased release of dopamine and norepinephrine in the prefrontal cortex (del Campo et al., 2011). Therefore, pharmacological treatments focus on correcting these neurochemical deficiencies. They can be broadly divided into stimulants (e.g., methylphenidate and dexamfetamine) and non-stimulants (e.g., atomoxetine, clonidine, and guanfacine) (Pliszka, 2003). Dexamfetamine- and methylphenidate-based medications are norepinephrine and dopamine reuptake inhibitors (NDRI) (Briars and Todd, 2016). Dexamfetamine based medications have the added action of increased release of dopamine. Atomoxetine is a selective norepinephrine reuptake inhibitor (SNRI), however, it also increases dopaminergic transmission in the pre-frontal cortex as norepinephrine reuptake is linked to dopamine inactivation (Fu et al., 2022). Clonidine and guanfacine are centrally acting alpha-2-agonists which decrease sympathetic outflow from the central nervous system. In the context of ADHD though their mechanism is not fully understood, the most popular theory is that these agents modulate post-synaptic alpha-2-receptors which translates into an enhanced pre-frontal cortex connectivity (Arnsten, 2010). Generally, stimulant ADHD medications are more effective at reducing the core symptoms of ADHD and are associated with greater effect sizes in terms of positive social and academic outcomes (Habel et al., 2011; Franke et al., 2018).

Starting in the 90s, concerns regarding the cardiovascular (CV) safety of ADHD medications were raised (Gutgesell et al., 1999). As a result, at the first clinic visit, it has become standard practice to take a CV history and, if appropriate, perform a CV examination including a resting 12-lead electrocardiogram (ECG), followed by heart rate (HR) and blood pressure (BP) measurements at the subsequent visits (Brown et al., 2018). Cardiology specialist opinion is often sought before commencing ADHD medications if there is a history of CV disease (CVD), sudden cardiac death (SCD) in a first relative, cardiac murmurs on examination, high heart rate or blood pressure, or abnormal ECG features [e.g., a long corrected QT interval (QT_*c*_)]. Although many cardiovascular diagnoses are not contraindications to commencing ADHD medications (e.g., valvular stenosis or regurgitation, atrial or ventricular septal defects etc.), the management in the context of a serious cardiac disease [e.g., hypertrophic cardiomyopathy (HCM)] is still a dilemma as guidelines often vaguely recommend a risk-benefit assessment. This topic is especially relevant since ADHD is more prevalent in patients suffering from congenital heart disease or cardiomyopathy (Mahle et al., 2000; Niarchou et al., 2015). Treating ADHD in these patients has been associated with heighted SCD risk (Vetter et al., 2008), while not treating leads to poor quality of life and other adverse outcomes (Barkley et al., 2006). Thus, there is a need to explore and quantify cardiovascular risks associated with ADHD medications.

## Review aim

The current review summarizes the cardiovascular complications and risks associated with ADHD medication, discusses their use in individuals suffering from cardiomyopathy and congenital heart disease and concludes with future research directions.

## Methodology

### Search process

In order to identify manuscripts in line with our review aim, a systematic search of Embase, PubMed, and Scopus was used to identify relevant publications published up to 01/10/2022. Search items were defined using the PICO (Patient/Intervention/Comparator/Outcomes) framework: (P) = (“Attention-Deficit Hyperactivity Disorder,” “ADHD”); (I) = (“ADHD medications,” “dexamfetamine,” “methylphenidate,” “atomoxetine,” “clonidine,” “guanfacine”); © = (“cardiovascular disease,” “electrocardiogram/ECG,” “hypertension/blood pressure,” “arrythmia/tachycardia/bradycardia/heart rate/long QT/prolonged QT/malignant ventricular arrythmia/ventricular arrythmia,” “cardiomyopathy,” “cardiac syndrome,” “heat failure,” “myocardial infarction,” “stroke/transient ischemic attack,” “aortic dissection/aneurysm,” “sudden cardiac death/sudden death/cardiac arrest”); (O) = (“outcome,” “risk,” “association”). The PICO framework categories were combined using “AND,” while we grouped the variations within categories via “OR.” Reference lists of included articles were also reviewed to identify further eligible publications.

### Inclusion and exclusion criteria and quality assessment

Inclusion criteria were peer-reviewed, English-written manuscripts available online through electronic indexing fulfilling the following criteria: (1) study participants had diagnosed ADHD formally diagnosed, (2) study participants received pharmacological ADHD treatment, (3) study participants had any form of cardiovascular disease, or the study aimed to highlight an association between ADHD medications and a cardiovascular complication. Non-original research (i.e., lecture notes, book chapters, conference abstracts etc.) were excluded. The quality of studies (e.g., risk of bias) was not formally evaluated through a validated tool, but papers were critically appraised in the discussion.

## Direct cardiovascular effects of attention-deficit hyperactivity disorder medications

The cardiovascular effects of medications used to treat ADHD in the general population are summarized in Table 1. Quantitative data relating the effects of ADHD medications on cardiovascular phenotypes based on previous studies and meta-analyses are presented in Table 2.

**TABLE 1:** The cardiovascular effects of medications used to treat ADHD in the general population.

Drug class	Drug name	Mechanism of Action	Heart rate	Blood pressure	Cardiac contractility	ECG changes	Pro-arrhythmogenic	Sudden cardiac death	MI or stroke
Stimulant	Amfetamine and derivatives	Sympathomimetic	**↑**	**↑**	**↑**	↔	AF, AT, AVNRT, AVRT, monomorphic VT	No	No
	Methylphenidate	NDRI	**↑**	**↑**	**↑**	↔	AF, AT, AVNRT, AVRT, monomorphic VT	No	No
Non-stimulant	Atomoxetine	Selective NRI	**↑**	**↑**	**↑**	**↔**	AF, AT, AVNRT, AVRT, monomorphic VT	No	No
	Guanfacine	α2-agonists	↓	↓	↓	**↔**	No	No	No
	Clonidine	α2-agonists	↓	↓	↓	**↔**	No	No	No

The cardiovascular effects are appraised based on either meta-analysis or clinical studies rather than case reports. Please note that these are applicable to the general pediatric population rather than those with established cardiovascular disease. Tricyclic antidepressants are no longer used in ADHD patients, so they are not presented in this table. AF, atrial fibrillation; AT, atrial tachycardia; AVNRT, atrio-ventricular nodal re-entrant tachycardia; AVRT, atrio-ventricular re-entrant tachycardia; ADHD, attention deficit hyperactivity disorder; ECG, electrocardiogram; MI, myocardial infarction; NDRI, norepinephrine dopamine reuptake inhibitor; NRI, norepinephrine reuptake inhibitor; VT, ventricular tachycardia.

**TABLE 2:** Quantitative data relating the effects of ADHD medications on cardiovascular phenotypes.

Phenotype	Study	Study type	*n*	Age group	Follow-up period	Aim	Qualitative CV findings
**General population**							
Heart rate and BP	Findling et al. (2005)	RCT follow-up	580	6–12 years	2 years	- Study effects of amfetamines on heart rate and BP in children	- After 2 years, mean increase in heart rate was 3.4 bpm, in SBP 3.5 mmHg and in DBP 2.6 mmHg with no dose-response relationship
	Mick et al. (2012)	Meta-analysis of 10 RCTs (Weisler et al., 2006; Biederman et al., 2007, 2012; Spencer et al., 2007; Adler, 2008; Medori et al., 2008; Adler et al., 2009; Rösler et al., 2009; Retz et al., 2012; Casas et al., 2013)	2,665	22–40 years	4–24 weeks (median 6 weeks)	- Study the effects of stimulants on heart rate and BP in adults	- Stimulants associated with a 5.7 bpm 95% CI (3.6, 7.8) higher heart rate and a 2.0 mmHg 95% CI (0.8, 3.2) higher SBP
	Wernicke et al. (2003)	Meta-analysis of 5 RCTs (Michelson et al., 2001, 2002, 2003; Spencer, 2002; Newcorn, 2002)	1,086 (536 adults)	All	Up to 10 weeks	- To study the effects of atomoxetine on heart rate and BP in children, adolescents, and adults	- Mean heart rate increased by 5–9 bpm across all ages, but there was no significant increase in SBP neither in children/adolescents nor in adults (*p* > 0.05)
	Vitiello et al. (2012)	RCT follow-up	579	7–9 years	10 years	-to study whether chronic use of stimulants associates with hypertension	- Stimulants were not associated with increased risk of pre-hypertension or hypertension (all *p* > 0.05)
	Huss et al. (2018)	RCT	215	6–18 years	2 years	- To study the long-term safety and efficacy of guanfacine in adolescents with ADHD	- At final assessment, the mean heart rate decreased by 5.5 (SD 13.0), SBP decreased by 0.6 (SD 9.3) and DBP (SD 9.2)
	Kisicki et al. (2007)	RCT	45	19–24 years	32 days	- To study the effect on BPP of abrupt cessation vs. tapper-down of guanfacine	- The taper-down and abrupt cessation groups exhibited statistically different elevations of neither SBP nor DBP
Major cardiac events	Habel et al. (2011)	Retrospective cohort study	443,398	25–64 years	21 years	- Evaluate the association between ADHD medications and serious CV events in adults	- The RR for the MI, stroke and SCD composite was 0.97 95% CI 0.84–1.12, and no significant result was found in individual analyses either
	McCarthy et al. (2009)	Retrospective cohort study	5,351	2–21 years	13 years	- Evaluate the association between stimulants/atomoxetine and SCD	- There was no increase in SCD associated with stimulants or atomoxetine (incident RR 1.63 95% CI [0.04, 9.71])
	Cooper et al. (2011)	Retrospective cohort study	1,200,438	2–24 years	Unclear	- Evaluated the association between ADHD medications and MI, stroke and SCD	- ADHD medication was not associated neither with a composite metric of MI, stroke and SCD HR 0.75 95% CI 0.31–1.85 nor with the individual components
	Schelleman et al. (2011)	Retrospective cohort study	241,418	3–17 years	180 days from the first dispensed ADHD medication	- Evaluated the rate of cardiovascular events and death in stimulants and atomoxetine users	- No statistically significant difference between ADHD medications and SCD or ventricular arrythmia HR 1.60 95% CI 0.19–13.60 or all-cause mortality HR 0.76 95% CI 0.52–1.12
	Gould et al. (2009)	Retrospective case-control study	1,128	7–19 years	Unclear	- To evaluate the association between stimulants and SCD	- Stimulant use associated with SCD OR 7.4 95% CI 1.4–74.9
	Liu et al. (2018)	Meta-analysis of observational studies (Gould et al., 2009; Cooper et al., 2011; Habel et al., 2011; Schelleman et al., 2011, 2012; Shin et al., 2016; ADHD Medications and Risk of Stroke in Young and Middle-Aged Adults, 2017)	4,221,929	All	Unclear	- To evaluate the association between stimulants and atomoxetine, and major cardiac outcomes	- There was no association between ADHD medications and a composite of SCD and arrythmia RR 1.24 95% CI 0.84–1.83, stroke RR 1.00 95% CI 0.74–1.35, MI RR 0.91 95% CI 0.79–1.05, or all-cause death RR 0.89 95% CI 0.54–1.45
	Mazza et al. (2013)	Meta-analysis of observational studies (Cooper et al., 2011; Habel et al., 2011; Schelleman et al., 2011)	1,884,963	All	806,162 person-years	- To evaluate the association between stimulants and atomoxetine and SCD	- ADHD medications did not increase the risk of SCD OR 0.93 95% CI 0.73–1.17, or stroke OR 0.93 95% CI 0.90–0.96

BP, blood pressure; CI, confidence interval; CV, cardiovascular; DBP, diastolic blood pressure; HR, hazard ratio; RCT, randomized controlled clinical trial; RR, rate ratio; OR, odds ratio; SBP, systolic blood pressure; SD, standard deviation; SCD, sudden cardiac death. Other abbreviations as in Table.

### Cardiac chronotropy, inotropy and systemic peripheral resistance

Stimulant drugs and atomoxetine are positively chronotropic and inotropic, and increase the peripheral systemic vascular resistance as they block the reuptake of norepinephrine and/or dopamine. Thus, they are associated with increases in HR, cardiac contractility, and BP (Tisdale et al., 2020). On the other hand, α2-agonists (clonidine and guanfacine) reduce the sympathetic outflow from the central nervous system having negative chronotropic and inotropic effects which is meditated by the reduced β1 stimulation in the heart. Similarly, the reduced α1 stimulation in the periphery is associated with a reduced peripheral vascular resistance which translates into a reduced blood pressure (Hermiller et al., 1983). Despite this, orthostatic hypotension is unusual (Strange, 2008) as the mean reduction in either systolic or diastolic BP is <5 mmHg (Huss et al., 2018). However, they are associated with a withdrawal syndrome when abruptly discontinued or weaned too fast which is thought to occur due to a rebound sympathetic hyperactivity (Van ZwiEten et al., 1984). This has been shown in approximately half of clonidine users in small sample studies having a total sum totaling less than 200 participants (Geyskes et al., 1979; Weber, 1980; Reid et al., 1984; Leckman et al., 1985), but only in 3% of guanfacine users which might be explained due the longer half-life of the later (Strange, 2008). To date, the risk factors, or the doses at which rebound hypertension can occur are yet to be established. Abrupt withdrawal of guanfacine has been shown to cause an increase from pre-treatment systolic BP values of up to 30 mmHg in adults with already established hypertension (Sorkin and Heel, 1986), but the only clinical trial (involving 45 healthy volunteers aged 19–24 years without hypertension) found no association (Kisicki et al., 2007).

### Electrocardiagram changes

An ECG is highly accessible screening test for CV disease. In certain conditions such as HCM, more than 95% have an ECG abnormality such as repolarization abnormalities (e.g., T-wave inversion, ST depression or elevation) in 80%, left ventricular hypertrophy (e.g., a high Sokolow-Lyon index) in 70%, or QRS-axis deviation in less than 40% (Norrish et al., 2021). However, the negative predictive value of a normal resting ECG is limited by conditions in which abnormalities are subtle (e.g., subclinical HCM; Captur et al., 2014) or only apparent during exertion (Amin et al., 2009). Still, it remains is a cost-effective screening tool (Fuller, 2000) and hence its widespread use (although not mandatory) before starting ADHD medications in clinical practice. However, none of the ADHD medications commonly used in practice bring about any apparent ECG changes (Vetter et al., 2008) besides the faster heart rate as per the scientific statement from the American Heart Association Council on Cardiovascular Disease in the Young Congenital Cardiac Defects Committee (Vetter et al., 2008). It should be noted that this conclusion was based on small clinical trials in stimulants (Dittmann et al., 2001; Findling et al., 2001), atomoxetine (Michelson et al., 2003; Wernicke et al., 2003), clonidine (Hazell and Stuart, 2003), or guanfacine (Hunt et al., 1995). Thus, the value of repeating the ECG for predicting who might develop an arrythmia or who is at risk of SCD is limited.

T-wave inversions in leads III and V4-V6, incomplete right bundle branch block and sinus arrythmia are common in the pediatric population (Norrish et al., 2021) and should not preclude the administration of ADHD medications. Premature atrial or ventricular contractions at low frequency and in the absence of cardiac disease, are also considered benign and should not prevent the initiation of ADHD pharmacological treatment (Picarzo et al., 2020).

### Pro-arrhythmogenicity

Both dexamfetamine and methylphenidate are pro-arrhythmogenic given their direct and indirect sympathomimetic effects. The increase in β-adrenergic stimulation of the heart may cause, precipitate or worsen atrial fibrillation (AF), supraventricular tachycardia [SVT; which includes atrial tachycardia (AT) and atrio-ventricular nodal re-entrant tachycardia (AVNRT)] or monomorphic ventricular tachycardia (VT) as per the American Heart Association consensus statement (Tisdale et al., 2020). The recommendations are based on retrospective cohort studies both in children (Dalsgaard et al., 2014; Shin et al., 2016) and adults (Habel et al., 2011; Schelleman et al., 2012) given the paucity of randomized controlled clinical trials (RCTs). However, this seems to be limited to the exposed time-periods only (Westover and Halm, 2012) with no evidence of long-lasting risks once the medication was stopped (Martinez-Raga et al., 2012). Although generally, increments in resting HR are associated with increased mortality in the general population (Hartaigh et al., 2014), it is unclear whether this holds true for drug-induced HR increases. In adults, although methylphenidate increases the risk of ventricular arrythmias, this effect was not dose-dependent in a cohort of 43,999 methylphenidate users which might suggest that this effect is not causal (Schelleman et al., 2012). In >100,000 person-years of observation using US Medicaid data, although stimulant use was not associated with cardiac death, an increase in emergency room visits but without an increase in hospitalizations was observed (Winterstein et al., 2007). Moreover, an insurance claim review of >150,000 participants found no association between stimulants and clinical symptoms of diagnoses of cardiovascular events (Olfson et al., 2012). Guanfacine and clonidine do not appear to be pro-arrhythmic (Martin et al., 2015; Constantin-Cristian Topriceanu, 2016) and clonidine has historically been shown to have HR-reducing properties in AF (Goodman, 1992) and to reduce the incidence of ventricular arrythmias in adults with heart failure (Zhang and Zhu, 1998). However, the studies which reported these findings are >30 years old with no recent studies replicating or refuting the claims.

#### Ancestry and attention-deficit hyperactivity disorder drug-response phenotypes

Variable drug-response phenotypes can be explained by differences in pharmacokinetics and pharmacodynamics (Yasuda et al., 2008; Schoretsanitis et al., 2019). The differences in pharmacokinetics arise mainly from the genetic variability of drug-metabolizing enzymes (Bjornsson et al., 2003) most of which belong to the CYP superfamily (Guengerich, 2019) which display a considerable inter-ethnic variability (Fricke-Galindo et al., 2016) with copy-number variants (i.e., more than two copies of functional alleles) leading to increased metabolism also being recognized (Ma et al., 2002). Amfetamines and atomoxetine are metabolized via a CYP2D6-mediated hydroxylation (Schoretsanitis et al., 2019), guanfacine is a substrate for CYP3A4 (Verplaetse et al., 2019), while clonidine is metabolized by both but mainly by CYP2D6 (Claessens et al., 2010). Based on the activity of the enzyme isoforms, patients can range from poor (prone to toxicity), intermediate, extensive/normal, rapid to ultrarapid (prone to reduce efficacy) metabolizers. Thus, poor and intermediate metabolizers are more likely to experience side-effects (e.g., tachycardia) (Kam and Jeong, 2020). For CYP2D6, Caucasians are more likely to carry the wild-type CYP2D6*1 allele and be normal (about 40%) rather than intermediate or poor (< 10%) metabolizers (Bradford, 2002; Ingelman-Sundberg et al., 2007). In contrast, CYP2D6*10 is common in Asians meaning that 30–40% are intermediate metabolizers (Gaedigk et al., 1999). In Africans, the percentage of functional alleles is around 50% with reduced function alleles (especially CYP2D6*17) being present in 30–40% of the population (Bradford, 2002). Similarly, there is an ethnicity related polymorphism in CYP3A4 (Guttman et al., 2019). Thus, pharmacogenomic biomarkers are an exciting prospect in the field of precision medicine and personalized pharmacotherapy (Kam and Jeong, 2020).

## Cardiovascular conditions requiring cautious attention-deficit hyperactivity disorder medication use

### Hypertension and tachycardia

As dexamfetamine can increase the BP, they are relatively contraindicated in ADHD patients with moderate and severe hypertension (Brown et al., 2018). However, the average increase in systolic and diastolic BP ranged between approximately 3–6 and 2–4 mmHg respectively, in clinical trials with a few hundred participants (Dittmann et al., 2001; Findling et al., 2001) (Michelson et al., 2003; Wernicke et al., 2003) (Samuels et al., 2006). Although some small studies reported an association between stimulant dose and BP effects (Stowe et al., 2002), adequately powered studies did not replicate the findings (Findling et al., 2005). Moreover, some of the acute effects on BP are attenuated with chronic treatment but full tolerance might not develop (Safer, 1992; Wilens et al., 2004; Hammerness et al., 2009). The Multimodal Treatment Study of Children with ADHD (MTA) RCT (NCT: 00000388) found no significant increase in systolic/diastolic BP in 579 children aged 7–9 over 14 months, and also concluded that stimulants were not associated with increased risk of pre-hypertension or hypertension over the 10-year naturalistic follow-up after the trial (Vitiello et al., 2012). Similarly, stimulant ADHD medication increase the heart rate by 4–5 bpm on average (Findling et al., 2005; Mick et al., 2012). The effects of atomoxetine are comparable to psychostimulants (Liang et al., 2018). Given that guanfacine and clonidine decrease the BP, their use in those with cardiac problems associated with hypertension has a double benefit. However, the mean reduction in either systolic or diastolic blood pressure is <5 mmHg in those who are normotensive as shown by clinical trials with a few hundred participants (Huss et al., 2018).

### Myocardial infarction, aortic dissection, and stroke

The association between amfetamines and vascular accidents such as myocardial infarction (MI) and hemorrhagic stroke became apparent from population studies based on drug users hospitalized patients (Westover et al., 2007; Huang et al., 2017). Although case reports of both exist, most studies did not find any associations between psychostimulant use and stroke or MI, neither in children and adolescents (Schelleman et al., 2011; Zito and Burcu, 2017) nor in adults (ADHD Medications and Risk of Stroke in Young and Middle-Aged Adults, 2011; Schelleman et al., 2012; Westover and Halm, 2012; Table 2). However, there might be a higher risk of MI but not for stroke in the first 2 months after starting methylphenidate as shown by a small study of 1,224 participants (Shin et al., 2016). In contrast, the increased risk of MI or stroke was not found to be significantly higher in those who recently started stimulants or atomoxetine in half a million participants (Habel et al., 2011). Moreover, most studies did not find any association between atomoxetine and MI or stroke (Holick et al., 2009; Schelleman et al., 2012; Houghton et al., 2020). However, stimulants/atomoxetine might increase the risk of a transient ischemic attack in adults (Holick et al., 2009) which was not observed for children.

### Coronary failure

Coronary failure is uncommon in children and adolescence. Often the causes include coronary artery anomalies (CAAs), acute Kawasaki disease and vasculitis (Graidis et al., 2015). The increased chronotropy reduces diastole when most blood flow through the coronary artery occurs. Moreover, the positive inotropic effect is associated with higher myocardial oxygen consumption. This is partially counterbalanced by the increased diastolic BP which promotes coronary blood flow. However, given the modest increase in both, neither should be significant in the context of normal physiology. However, in those with coronary failure psychostimulants and atomoxetine can theoretically lead to myocardial supply-demand mismatch which predisposes to a myocardial infarction. As above, this is especially relevant in the period immediately after starting the ADHD medication. Similarly, by decreasing the blood pressure, α2-agonists also reduce coronary perfusion. Thus, all ADHD medications pose theoretical list in those with coronary failure. In acute conditions such as Kawasaki disease and vasculitis, holding the ADHD medications until resolution would be the safest approach. CAAs describe a spectrum from almost normal to completely abnormal coronary blood flow (Graidis et al., 2015). Despite no clinical data, use of sympathomimetic drugs in those with moderate-severe CAAs should be cautious.

### Aortopathies

Aortic diameter above 3.5 cm is considered to be dilated in adults, but in children normograms with height or body surface area indexation are used. The greater the dilatation, the more likely it is to rupture or to dissect (Erbel and Eggebrecht, 2006). In patients with a dilated proximal aorta, e.g., in the context of Marfan’s syndrome, increases in BP can further exacerbate the dilatation of aorta which might be prone it to rupture (Singh et al., 2021). Thus, psychostimulants and atomoxetine should be avoided, but α2-agonists are safe. Nevertheless, many of those who have aortic dilation are usually already on BP lowering therapy such as β-blockers meaning that they might not tolerate the further drop in BP brought on by the α2-agonists.

### Patients at risk of sudden cardiac death

Excluding sudden infant death syndrome (SIDS), the commonest causes of sudden cardiac death in children are: (1) structural cardiac disease (e.g., CAAs), (2) cardiomyopathies [HCM, dilated cardiomyopathy (DCM)], (3) arrhythmic cardiomyopathy (AC) and (4) channelopathies (e.g., long QT, Brugada syndrome) with HCM and CAAs being the top two causes (Gajewski and Saul, 2010). In the general pediatric population, studies have obtained mixed effects regarding the association of psychostimulants and atomoxetine with SCD. Some large studies with a combined sample size of almost two million participants found no association (McCarthy et al., 2009; Cooper et al., 2011; Habel et al., 2011) while relatively smaller studies of a quarter of a million participants reported 2–7 times higher odds of SCD (Gould et al., 2009; Schelleman et al., 2011), although they did not adjust for confounders (Schelleman et al., 2011) or failed to address selection bias (Westover and Halm, 2012). These associations were not significant in meta-analyses but the uncertainty arising from the wide confidence intervals of the hazard ratios (Mazza et al., 2013; Liu et al., 2018) highlight the need for more research to understand the association between ADHD medications and SCD.

### Hypertrophic cardiomyopathy

HCM is relatively common affecting 1 in 500 adults and is the leading cause of SCD in the developed world. Current predictors of SCD in HCM include family history of SCD, a left ventricular outflow tract obstruction (LVOTO), high maximal wall thickness (MWT) and non-sustained VT (NSVT) (Norrish et al., 2021). However, these high-risk patients often have implantable cardioverter defibrillations (ICDs) inserted which allows for a more liberal use of ADHD medications (Kantor et al., 2021).

By lowering the blood pressure, α2-agonists increase the LVOT gradient, but this is counterbalanced by their negatively inotropic effect which decreases any LVOTO (Reza et al., 2021). Although the degree of LVOTO is directly related to the risk of SCD (Elliott et al., 2006), minor dynamic increases might not be clinically significant. Moreover, clonidine is considered safe in HCM and is sometimes used to treat hypertension in these patients (Argulian et al., 2013; Sherrid, 2016). The decreased sympathetic outflow brought about by α2-agonists could be beneficial in HCM extrapolating from the use of β-blockers (Hensley et al., 2015).

By increasing adrenergic conduction, psychostimulants and atomoxetine can predispose to NSVT and other atrial and ventricular tachyarrhythmias, and therefore are generally avoided in HCM. Although this is the consensus, it is based mostly on biological plausibility, case reports and retrospective cohort studies (Dalsgaard et al., 2014) with no RCTs being published.

### Dilated cardiomyopathy

Although in adults, DCM is often secondary to ischemic heart disease, hypertension, and excessive alcohol (Reichart et al., 2019), it is commonly genetic in the pediatric population (Lee et al., 2017) or in those with a positive family history. The association between amfetamines and cocaine use and the developmental of DCM from cardiotoxicity is well established (Lange and Hillis, 2001). As excess catecholamines are toxic to the heart, stimulants could potentially lead to DCM. Although theoretically possible, the association of DCM with psychostimulants at therapeutic doses in ADHD is limited to case reports (Nymark et al., 2008) with no association with heart failure being seen in larger studies (Shin et al., 2016). In those with already established DCM, the use of sympathomimetics is controversial given the increased myocardial workload (Page et al., 2016). However, α2-agonists decrease the heart rate and the afterload and could be beneficial (Hermiller et al., 1983; Girgis et al., 1998) unless the blood pressure is already low. In that scenario, they could cause orthostatic hypotension, syncope, or even hemodynamic collapse.

### Long-concerns regarding syndrome

In the general pediatric population, none of the ADHD medications have been associated with torsade de points (i.e., polymorphic VT) or significant increases in QT_c_. This also holds true for the use of stimulant medications in patients with long-QT syndrome (*n* = 144) although higher odds for syncope was demonstrated (Zhang et al., 2015). Extrapolating from use of β-agonists in asthma, the syncope was postulated to be secondary to arrythmias as those with long-QT syndrome have lower threshold to experience them when exposed to sympathomimetic agents (Thottathil et al., 2008). Although atomoxetine reduces K^+^ efflux from the heart as an antagonist of I_Kr_ channel (Scherer et al., 2009), this is not problematic in the general pediatric population at a therapeutic dose as it is not associated with a prolonged QT. However, at supratherapeutic doses the QT_c_ is increased (Martinez-Raga et al., 2012) in a dose dependent fashion (Loghin et al., 2013). As I_Kr_ is implicated in long-QT syndrome, use of atomoxetine in those with long-QT syndrome is theoretically problematic, but this has not been evaluated in clinical studies. Although there is a warning from the European Medicines Agency (EMA) regarding the prospect of α2-agonists (e.g., guanfacine) use in those with long-QT syndrome, clinical studies concluded that they do not interfere with cardiac repolarization and are not known to be pro-arrhythmic (Martin et al., 2015; Constantin-Cristian Topriceanu, 2016) and hence this warning might be overly-cautious.

### Brugada syndrome

Besides blocking the cardiac I_Kr_ channel (Scherer et al., 2009), atomoxetine can also block the hNa_v_1.5 channels at therapeutic levels (Föhr et al., 2021) which makes it a potentially unwise choice for use in children suffering with Brugada syndrome (Brugada et al., 1999) as it can potentially push the heart into ventricular fibrillation (VF). For the same reason the use of TCAs is contraindicated. However, neither stimulants nor α2-agonists were shown to interfere with cardiac Na^+^ channels. Since the stimulants increase the adrenergic transmission and further increase the risk of arrythmias (Tisdale et al., 2020), there is an argument for avoiding them in Brugada syndrome. However, there are no reported contraindication to α2-agonists based on the literature (Francis and Antzelevitch, 2008). Nevertheless, the use of any ADHD medications continues to be commonly avoided in patients suffering from ADHD and Brugada syndrome (Gundogmus et al., 2018) depriving them from adequate ADHD management.

### Wolf-Parkinson-White syndrome

According to the AHA consensus statement, ADHD medication can be started in those with Wolf-Parkinson-White (WPW) syndrome, but close monitoring is warranted. The prospect of developing atrio-ventricular re-entrant tachycardia (AVRT) is linked to atrio-ventricular node blockade as it is the case with β-blockers (Babayiğit et al., 2020). As psychostimulants and atomoxetine increase the sympathetic drive in the heart, they have not been classically associated with AVRT despite being associated with AVNRT (Tisdale et al., 2020). However, they might promote AF (Tisdale et al., 2020). If that happens, the rate-limiting effects of the AV node are bypassed, and conduction occurs through the accessory pathway potentially leading to VF and cardiac arrest. Although there is no study which reported the incidence of AF in children/adolescents treated with psychostimulants and atomoxetine, it seems to be a rare phenomenon mainly seen in case reports (Gao et al., 2020). Thus, avoiding them in WPW might be overly cautions. However, by decreasing the sympathetic outflow to the heart, α2-agonists can reduce the conduction through the AV node (Kibler and Gazes, 1977) potentially predisposing to AVRT. However, this is more a theoretical risk as no reports of α2-agonists being associated with AVRT or SCD in WPW were found. Furthermore, WPW can be cured through electrophysiological ablation which would allow a more liberal use of ADHD medications in these patients (Shetty et al., 2011).

## Conclusion

Attention-deficit hyperactivity disorder treatment has been shown to decrease antisocial behavior, poor academic performance, and mortality but to date, many individuals with cardiovascular disease continue to be deprived of ADHD medications because of overly cautious approaches unsupported by the evidence-base. Review of published evidence shows that stimulant and non-stimulant ADHD medications have generally good cardiovascular safety profiles but given their mechanisms of action, they should be used cautiously in children and adults with pro-arrhythmic cardiovascular diseases. Although α2-agonists are not as effective as stimulants, they are not pro-arrhythmic and therefore safer in those at higher risk of arrhythmias. The increase or decrease in blood pressure associated with stimulants and α2-agonists, respectively, is not usually clinically significant so clinicians should not be deterred from starting them although their abrupt withdrawal, especially of clonidine, can be problematic.

## Ethics statement

All procedures performed were in accordance with the ethical standards of the institutional and/or national research committee and with the 1964 Helsinki declaration and its later amendments or comparable ethical standards. As the data used in this manuscript is publicly available, an ethics approval waiver was applied.

## Author contributions

C-CT guarantor of this work and attested that all listed authors meet the authorship criteria and that no others meeting the criteria have been omitted. All authors contributed significantly to the study design and implementation, data analysis and interpretation, and manuscript writing, involved in critically reviewing and revising the manuscript, approved the final version as submitted, and agree to be accountable for all aspects of the work.
